# Relevance of Tibial Fixation during Tibiotarsal Joint Traction: Descriptive Cross-Sectional Study

**DOI:** 10.3390/jfmk9030163

**Published:** 2024-09-15

**Authors:** Carlos López-de-Celis, Jacobo Rodríguez-Sanz, Sergi Gassó-Villarejo, Erik García-Ribell, Vanessa González-Rueda, Elena Estébanez-de-Miguel, Elena Bueno-Gracia

**Affiliations:** 1 Department of Physiotherapy, Faculty of Medicine and Health Sciences, Universitat Internacional de Catalunya, 08195 Barcelona, Spain; carlesldc@uic.es (C.L.-d.-C.); sergi.gasso@uic.es (S.G.-V.); erik.garcia@uic.es (E.G.-R.); 2 ACTIUM Research Group, Universitat Internacional de Catalunya (UIC), 08195 Barcelona, Spain; vgonzalezr.apms.ics@gencat.cat; 3 Institut Universitari d’Investigació en Atenció Primària (IDIAP Jordi Gol), 08007 Barcelona, Spain; 4 Department of Medicine, Faculty of Medicine and Health Sciences, Universitat Internacional de Catalunya, 08195 Barcelona, Spain; 5 Health Sciences Faculty, Department of Physiatry and Nursing, University of Zaragoza, C/Domingo Miral S/N, 50009 Zaragoza, Spain; elesteba@unizar.es (E.E.-d.-M.); ebueno@unizar.es (E.B.-G.)

**Keywords:** ankle joint, manual therapy, mobilisation, ultrasound

## Abstract

**Background:** The effect of tibial fixation on the movement of the talus during the tibiotarsal axial traction technique (TATT) is unknown. The aim was to evaluate the effect on the tibiotarsus when applying three different intensities of TATT force with or without tibial fixation in healthy subjects, and to assess the reliability of detecting the different forces applied. Also, the discomfort generated during the technique would be analysed. **Methods:** A cross-sectional study was conducted in thirty lower limbs. Three magnitudes of TATT force in an open-packed position were applied in tibial fixation and non-fixation conditions. The axial traction movement was measured by ultrasound, and the magnitudes of the force applied during low-medium and high TATT force were recorded in both conditions. Patients were asked about the level of discomfort perceived during the technique. **Results:** The most significant distance increase (mm) was observed in the tibial fixation condition at all magnitudes of the TATT (F = 102.693, *p* < 0.001). The discomfort sensation (numeric rating scale, “NRS”) was higher in the non-fixation condition (*p* > 0.05). The application of the technique showed good reliability (>0.75 ICC) for the detection of the applied force. **Conclusions:** The TATT in the tibial fixation condition produced more significant axial movement of the talus (mm) relative to the tibia than the non-tibial fixation condition did. The detection of the magnitudes of movement showed good reliability (ICC: 0.75 to 0.92). The technique was well tolerated at all force magnitudes, with the tibial fixation condition being the most tolerable.

## 1. Introduction

Traumatic ankle injuries are common and are not always associated with sports activities. Fractures of the ankle joint have an incidence of up to 174 cases per 100,000 people per year [1], with lateral malleolus fractures accounting for 55% of fractures [2]. A review by the National Trauma Data Bank between 2007 and 2011 found 280,933 fracture dislocations during this 4-year period [3]. Another pathology affecting the lower extremities is ankle sprains, which have a higher incidence in women than in men (13.6 vs. 6.94 per 1000 exposures) [4]. High-quality studies estimate an incidence of 11.55 per 1000 exposures [4], with a high recurrence rate that is associated with the development of chronic ankle instability [5,6]. They are also associated with later-life joint disorders [7,8].

Chronic musculoskeletal problems after immobilisation [9,10] are known, but we must also consider pain secondary to immobilisation or joint compression [11,12]. These lower extremity musculoskeletal injuries can cause short-term disability and interfere with returning to work and physical activity [13,14].

In the recovery process after immobilisation, the priorities are to restore joint mobility, pain control and function recovery. There are different methods with slight variations. The Mulligan method proposes mobilisation with movement [15,16], while Maitland proposes the passive use of gentle passive mobilisation [17] and Kaltenborn a translational movement for joint mobilisation [18]. It is a widely used technique presenting minor differences in some nuances, such as the fixation or non-fixation of the proximal segment [19,20,21,22]. We can also find it as talocrural joint mobilisation, although we consider that the tibiotarsal axial traction technique (TATT) refers more to what we produce [19]. Therefore, we will describe the translatory traction technique according to Kaltenborn [18]. The manual traction mobilisation technique is used to increase the range of motion. This technique applies a linear, non-rotational joint movement, increasing tension in all the capsuloligamentous structures, increasing joint mobility and decreasing symptoms [23,24]. Some authors suggest that the decreased joint pressure and changes in capsular elasticity and surrounding muscles caused by joint distraction would explain the improved mobility and analgesic effects after applying manual techniques [23,25,26]. The response in the capsuloligamentous tissue would depend on the traction force applied in the joint mobilisation [27,28]. There would, therefore, be a relationship between the magnitude of the force applied and the resistance to the tensile force.

The translatory traction technique can be performed with different intensities of force application (low, medium and high), known by Kaltenborn as grades I, II and III. Low force (before eliminating the joint slack) is indicated for pain reduction [24], and medium force (tightening the periarticular tissue until you feel the maximum resistance to movement, called “first stop”) is used both for symptom relief and to begin to gain mobility. Finally, high force (applied beyond the first stop) is used to improve function and movement gain. [23,24]. These effects are modulated by the magnitude of the force applied or the degree of mobilisation [23,24]. The structural and cellular changes estimated in the ligament modify the tissue’s mechanical properties, increasing the joint’s range of motion [29,30].

In addition to its therapeutic objective, the tibiotarsal axial traction technique (TATT) is also used in magnetic resonance imaging (MRI) studies for the morphological evaluation of cartilage [31,32] and in arthroscopic ankle surgery [33,34]. In both techniques, a strap is used to maintain sustained traction on the foot and cause a separation of the articular surfaces. The main difference between these techniques and the therapeutic use of the TATT lies in the application time, which is much shorter for the treatment technique [35].

The axial traction technique can be applied to other joints. Its effect has been previously analysed in the hip joint in cadavers [27,36,37], showing that it is a specific technique for the capsule and finding an association between the force applied and the separation of the hip joint, also producing an increase in the range of motion (ROM). In living subjects, the effects of axial [38] and lateral traction [39,40] have also been studied at the glenohumeral level. In both cases, it has been observed that performing proximal bone fixation produces more specific effects on the joint to be moved. The same force produces a greater separation of the articular surfaces when proximal bone fixation is performed compared to the technique without such fixation [38,40].

Similar techniques with high velocity [20,21,41,42] and mobilisation [35] are performed at the tibiotarsal joint. The previous literature shows that these techniques should be performed with tibial fixation [21] to concentrate the effect of the traction on the tibiotarsal joint. However, we can also find them without tibial fixation [20,21,41,42]. This is an aspect that, to date, has not been studied in the TATT. 

We have not found any studies evaluating the effect of tibiotarsal traction during the application of low, medium and high force with and without tibial fixation, as well as the effect perceived by the patient during the application of the tibiotarsal axial traction technique (TATT). Our study hypothesises that the TATT with fixation has more of an effect on tibiotarsal joint distraction when the therapist applies a similar force in healthy subjects. The therapist is also reliable in detecting the force at all three magnitudes. The tolerance to the technique by the subject is similar in both fixation conditions. Therefore, the aim of this study was as follows: (1) to evaluate the effect on tibiotarsal joint traction during the application of the tibiotarsal axial traction mobilisation technique at the three magnitudes of force (low, medium and high) in the two clinical application conditions (tibial fixation and tibial non-fixation) in healthy subjects; (2) to know the force applied for each magnitude of force; and (3) to evaluate the discomfort produced when applying the TATT in both conditions.

## 2. Materials and Methods

### 2.1. Study Design and Ethics

A cross-sectional study was conducted. A repeated measures design was used to analyse the axial movement of the talus while applying three magnitudes of TATT force (low, medium, high) across two conditions (tibial fixation or tibial non-fixation). The study was conducted on a sample of subjects without tibiotalar pathology.

The primary study variable was the axial movement of the talus measured by ultrasound. The secondary variables were the magnitude of the force applied during low, medium and high TATT and the tolerance of the subjects during and after the application of the TATT.

This study was carried out with the ethical approval of the Clinical Research Ethics Committee for Medicines (CEIm-UIC. FIS-2023-07). The procedures followed were in accordance with the Declaration of Helsinki 1975, revised Fortaleza 2013.

The statistical power of the tibiotarsal distraction was calculated with G-power v3.1, reporting a statistical power of 99% for the high-force magnitude, which would require the most fixation. We would have had to include four joints in each group at the high-force magnitude.

### 2.2. Sample

The sample, thirty lower limbs from fifteen healthy subjects, was recruited from the Universitat Internacional de Catalunya. The inclusion criteria were the following: subjects over 18 years of age who signed the informed consent. The exclusion criteria were the following: (1) a history of orthopaedic injuries in the tibiotarsal joint that required surgical intervention, (2) presenting pain in the tibiotarsal joint, (3) a history of orthopedic injury in the lower extremity under study (fractures, grade II or III sprains), (4) a history of sprain (grade I) that has required treatment in the last 6 months, (5) a diagnosis of ligamentous hypermobility or Ehlers–Danlos syndrome.

### 2.3. Experimental Procedure

The procedure was performed in a single session. First, the inclusion and exclusion criteria were checked. Subsequently, sociodemographic characteristics were recorded: age, sex, height, weight and body mass index. 

**Figure 1:**
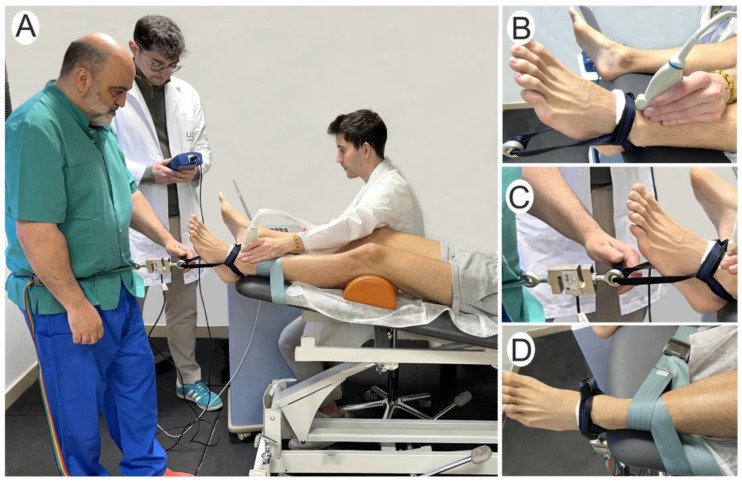
Experimental set-up. (**A**): Tibiotarsal axial traction technique in fixation condition; (**B**): ultrasound transducer position; (**C**): tibiotarsal traction strap and digital force gauge; (**D**): tibial fixation.

A single physical therapist with more than 25 years of clinical experience performed all TATTs. A second physical therapist with more than 8 years of experience in musculoskeletal ultrasounds performed all ultrasounds. A third evaluator recorded the magnitude of the force applied during the low-, medium- and high-force TATTs. This same evaluator asked for the tolerance of the TATT force in both conditions from the studied subjects (Figure 1A).

First, the tibial fixation condition was applied. The subject was in the supine position with the foot to be treated outside the table, with a cushion about 10 cm thick under the knee. The tibia was fixed with a strap on the distal part of the tibia above the malleolus (Figure 1B).

Once the tibial strap was fixed, the tibiotarsal traction strap was placed, which collects the posterior part of the calcaneus and the talus. A slight padding was placed between the strap and the subject’s skin to prevent skin injuries (Figure 1C).

For the TATT, the mobilising physical therapist placed a mobilisation strap around his pelvis. This mobilisation strap was attached to the tibiotarsal traction strap, and a dynamometer (475055 Digital Force Gauge; Extech, Boston, MA, USA) was placed between them to measure the magnitude of the applied force (low, medium and high) (Figure 1C).

The physical therapist who applied the TATT was unaware of the magnitude of the force exerted or the ultrasound images. The third physical therapist recorded the amount of the force applied from the dynamometer data (Figure 1A–C).

The tibiotarsal joint was in an open-packed position (10° of plantar flexion and mid-position between inversion and eversion) [18]. The subjects were instructed to keep their lower extremities relaxed. 

**Figure 2:**
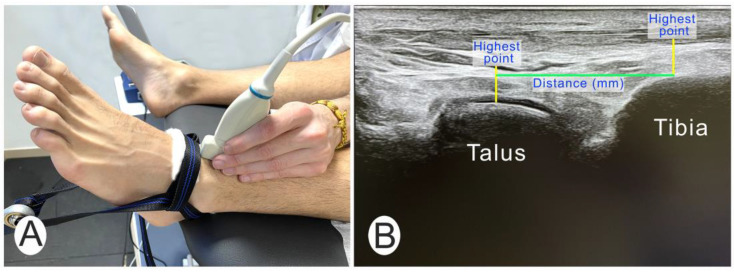
(**A**). Transducer position at anterior part of ankle joint. (**B**): Ultrasound image of tibia and talus at rest as baseline.

For the ultrasound measurement, a 40 mm linear transducer (USTTL01, 12L5) from a portable ultrasound machine (US Aloka Prosound C3 15.4) was placed longitudinally on the ventral and slightly medial aspect of the tibiotarsal joint, locating the joint interline (Figure 2A). The transducer was moved until the joint line was visible on the ultrasound image. Ultrasound measurements were taken at the different magnitudes of force when applying the TATT. In each image, the tibiotarsal distance was drawn, and considered the shortest line (mm) from the most distal upper point of the tibia to the perpendicular of the highest point of the talus (Figure 2B).

The TATT was performed by a physiotherapist, who axially pulled the ankle at three different magnitudes of TATT force according to the Kaltenborn degrees of joint mobilisation. The ankle joint was set in an open-packed position [18]. An ultrasound image was taken at zero force (baseline) as a reference, and subsequently, ultrasound images were taken during the application of low, medium and high force. The images were saved in the ultrasound machine itself and measured later.

Another ultrasound image was taken when the physical therapist determined that each of the three magnitudes of force or grades was reached. The low-force TATT point was determined when the physical therapist verbally indicated that the joint looseness had been reduced. The medium-force TATT US image was taken when marked resistance was first felt (the “first stop”) [18], and the high-force TATT was taken when the maximum tissue resistance occurred. This procedure was applied in the same sequence and repeated twice, with a 30-second rest between the repetitions. The recording values of the different magnitudes of the force applied in each repetition were used to assess the reliability of the physiotherapist who performed the TATT. 

Once this condition was completed, the same procedure was applied with the tibial non-fixation condition. The subject maintained the supine position with the tibiotarsal resting position. A zero-force (baseline) ultrasound image was taken. The magnitude of the force applied during the low, medium and high TATTs in the tibial non-fixation condition was the average value recorded during the tibial fixation condition, such that the tractions were the same intensity as in the fixation condition. The mobilising therapist performed the traction until the third physical therapist verbally indicated that the mean values of the low, medium and high forces had been reached. In this way, the ultrasound image was captured at the different magnitudes of force applied. The procedure was repeated a second time in the non-fixation condition. The average distance recorded in the two trials was used for the statistical analysis.

### 2.4. Reliability of Ultrasound Measurements

In addition, two evaluations were performed on five subjects with the same characteristics as the study sample before the study to determine the intra-rater reliability of the ultrasound image measurements. The aim was to determine whether the person performing the measurements was reliable enough to guarantee the results of the ultrasound measurements [40,43]. The intraclass correlation coefficient (ICC3,1) (two-way mixed effects model) the standard error of measurement (SEM), and the minimum detectable change at the 95% confidence level (MDC95%) for the ultrasound measurements of the axial movement of the talus to the tibia are shown in Table 1. For the interpretation of ICC3,1, values less than 0.5 indicate poor reliability, values between 0.5 and 0.75 indicate moderate reliability, values between 0.75 and 0.9 indicate good reliability, and values greater than 0.90 indicate excellent reliability [44].

To evaluate the feeling of discomfort during the application of the TATT, the subjects were asked about the discomfort level of the technique at the different magnitudes of applied force and in each fixation condition. For this, the “numeric rating scale” was used [45,46], ranging from 0 to 10 points. Zero is the minimum discomfort value, and ten is the maximum value. The NRS is a scale that has already been used for assessing discomfort [46,47], where discomfort is the painful sensation exerted by the application of the technique. The subjects were asked the perceived discomfort at each magnitude of the force applied.

### 2.5. Statistical Analysis

IBM SPSS Statistics for Windows, Version 20.0. (Armonk, NY, USA: IBM Corp.) was used for all statistical analyses. Descriptive statistics (mean and standard deviations, or number and percentage) were calculated to describe the sample’s demographic characteristics. For the comparative analysis of the differences in the axial movement of the talus movement with the magnitude of the TATT, the two-way ANOVA 2 × 3 (fixation condition x increase in distance) test was used. In the case of finding an interaction, the 2 × 3 analysis was performed with the Bonferroni correction [48]. The effect size was calculated to estimate the magnitude of the difference between the two conditions on the main variables with Cohen’s coefficient (d). The Cohen’s coefficients were interpreted as follows: large effect sizes, d > 0.8; moderate effect sizes, moderate, d = 0.5–0.79; and small, d = 0.2–0.49 [49]. 

## 3. Results

Regarding the prior evaluation of the reliability of the measurements, the results are shown in Table 1.

Thirty tibiotarsal joints from fifteen voluntary subjects (eight male, seven female) were examined in the fixation and non-fixation conditions. The demographic characteristics of the sample are shown in Table 2.

Table 3 shows the distance achieved during the application of the TATT at the three force magnitudes, as well as the differences between the tibial fixation and non-fixation conditions.

The two-way repeated measures ANOVA showed a significant interaction (F = 102.693 [*p* < 0.001]), for the increase in the distance (F = 375.320 [*p* < 0.001]) and condition (F = 69.997 [*p* < 0.001]). Statistically significant differences were found at all force magnitudes in the axial movement of the talus to the tibia between the fixation and non-fixation conditions with the Bonferroni post hoc test (Table 3).

The more significant axial movements of the talus to the tibia occurred in the tibial fixation condition at the three TATT force magnitudes. The most significant mean difference, 2.0 mm, was found in the distraction of the talus to the tibia during the high-force TATT.

Additionally, the inter-observer reliability of the applied force at the different magnitudes was analysed in the entire sample between the first and second applications (Table 4). The reliability of the low force was moderate, while the medium and high forces had good reliability.

Regarding the discomfort during the application of the three magnitudes of TATT force in the two conditions, the low force in the two conditions showed a mean value of zero. For the medium force, the mean value was 1.0 ± 1.2 for the fixation condition and 1.2 ± 1.3 for the non-fixation condition. For the high force, the mean values were 3.7 ± 2.3 and 4.1 ± 2.3 for the fixation and non-fixation conditions, respectively. No statistically significant differences existed between the fixation and non-fixation conditions at any of the force magnitudes.

## 4. Discussion

To our knowledge, this is the first study that analyses the effect of tibial fixation on the axial movement of the talus relative to the tibia by applying three different magnitudes of forces during the TATT. The results of the present study show that the axial motion of the talus was significantly greater in the tibial fixation condition for all three TATT force magnitudes in healthy subjects. This result is similar to another study that evaluated the effect of fixation on lateral glenohumeral mobility [40]. The reliability the force required for the different magnitudes was good for all force magnitudes. These results are similar to studies that also evaluated the effect of glenohumeral joint fixation [38,40]. In addition, tolerance to the technique was good, showing no differences between the fixation and non-fixation conditions. All of this leads us to accept the initial hypotheses raised in the manuscript.

The TATT is widely used with high [20,21,41,42] and low speed application [35] to increase the tibiotarsal joint ROM and/or improve pain. Therefore, clinically it should be performed with fixation of the tibia [21], to concentrate the effect of the traction on the tibiotarsal joint. Tibial non-fixation does not concentrate the force applied throughout the limb, minimising its effect. This has been confirmed in the most significant effect found in the fixation condition with a similar applied force. However, we can find authors who perform it without tibial fixation [20,21,41,42].

The ultrasound measurements showed that the axial movement of the talus relative to the tibia at all magnitudes was higher in the fixation condition. In MRI studies, sustained traction with a traction force is applied to separate the articular surfaces and better visualise the joint. A weight between 8 and 10 kilograms is used to perform the test, and this force is similar to that of other MRI studies [31,32,50]. This weight is lower than the average force magnitude used in our study, achieving a separation between 0.6 mm [31] and 1.2 mm [32], which is also a distance smaller than what we have found in our study. Because the performance of the MRI test is similar to that used in the present study, the greater separation of the articular surfaces obtained in this study could be related to the joint position used. In the present study, traction was performed in the joint rest position, a position in which the capsule and ligaments are more relaxed [18] and allow greater movement of the joint.

The lower force applied in these testing and imaging or arthroscopy studies may be due to the duration of the technique with sustained force, since a force greater than 35 pounds (155.6 N) can produce paresthesias in the posterior tibial nerve and in the deep peroneus [34,51]. This force magnitude is similar to the average force in our study. The difference is that the TATT applies force rhythmically and for shorter durations, thus making the technique more tolerable. As can be seen in the discomfort results, the maximum discomfort scores were an average of 3.7 and 4.1 points out of 10 during the high-force TATT.

Other studies that have analysed the effect of traction on other joints have obtained values of 0.79 mm to 3.72 mm of separation of the articular surfaces, similar to those obtained in this study [40,52]. This may be due to the greater mobility of the joint. In another study of glenohumeral axial traction with a maximum application force similar to this study, somewhat higher values were achieved [38].

The tibial fixation or non-fixation conditions were randomised, using a methodology similar to that of other studies [38,40]. However, a possible stretch accumulation effect, which could have led to greater distances as the techniques were performed, did not occur. The non-fixation values carried out with the average force applied in the fixation condition produced lower distraction values, so this aspect did not influence the measurements.

The distance achieved at low force, similar to MRI studies [31,32] where tension is exerted to visualise structures without compression, would corroborate that the application of a low force of the TATT is well described as eliminating intrinsic tension forces.

The reliability of the detection of medium and high force was good but not excellent (ICC = 0.849–0.896), surpassing the SEM at all magnitudes. The reliability of the detection of the degrees of movement has already been evaluated in other studies, showing good or excellent intra-observer reliability [23,37,53,54,55,56,57]. The differences between studies may be due to the characteristics of the joint being studied or to the difference in the harness used in the experiment. These forces represent a clear stop in the tensioning of the tissue, so the greater reliability in their detection does not coincide with low force.

Following Kaltenborn’s indications [18], the low-force and medium-force traction techniques are used for pain relief. This agrees with the tolerance that the subjects have expressed about the technique, with a value of 0.0 for the low force and 1.2 for the medium force. The high force is used to achieve joint traction and tissue elongation [58,59,60], which tightens the tissue and decreases its tolerance. The most significant discomfort value was 4.1 for the high force in the non-fixation condition; probably, the harness contributed to these magnitudes of force, and the condition does not allow a more specific focus of the technique. Even so, this is an acceptable value for a therapeutic technique that is clinically applied with the hands, so it is a clinically accepted improvement. However, it was not measured to which specific structures this tension was attributed. Studies similar to those carried out by Estébanez-de-Miguel et al. [27,36,37] in the hip region would be necessary to know the amount of stress received by the different structures and its relationship with the force applied during mobilisation. 

It is known that differences in muscle activation capacities [61,62], anthropometric variables [63] and flexibility [64] according to sex may exist. The purpose of the study was not to analyse these differences. However, to avoid this influence, we tried to ensure that the sample was homogeneous for this variable. In addition, it was decided not to take subjects with pathology as they may present a defense mechanism for fear of reproducing their injury or presenting painful symptoms, which could activate a defense response that would bias the results.

In clinical practice, when patients with limited tibiotarsal movement are treated with the TATT, as may be the case for subjects who have required immobilisation, it would be advisable to fix the tibia to provoke a specific axial traction movement of the talus, achieving a more significant distraction effect on the joint. It would also be indicated in those patients who may present with concomitant pathology in the knee or hip since, in this way, the effect is focused on the tibiotarsal joint, minimising the transmission of force to other joints. Ultrasound measurements are a reliable tool for evaluating the joint distraction [53,54]. The present study observed excellent intra-observer reliability (ICC greater than 0.99), superior to the SEM at all force magnitudes.

The main limitation of this study is that the values for strength, distance and tolerance correspond to a sample of healthy subjects, so they are likely to differ in subjects with pathology. Although they were given the order to remain relaxed, we cannot be sure that a contraction of the defense occurred, modifying the actual values. Our study cannot guarantee that these results are comparable by sex since it is a small sample, and a segmented analysis by sex was not performed. Furthermore, it would be indicated that studies be carried out in patients who present pathologies with mobility restrictions to evaluate whether the force applied to the degrees of movement corresponds to what is clinically attributed.

## 5. Conclusions

The TATT in the tibial fixation condition produced more significant axial movement of the talus with respect to the tibia than the non-tibial fixation condition did. The detection of the degrees of movement showed good reliability for all magnitudes of force. The technique is well tolerated at all magnitudes of force, and it is in the condition of tibial fixation where the greatest tolerance exists.

## Figures and Tables

**Table 1:** Outcomes of ultrasound measurements of axial movement of talus during three magnitudes of TATT force.

Magnitude of Force	ICC_3,1_	95% CI	SEM	MDC_95_
Baseline	0.996	0.968–1.000	0.12	0.34
Low-force TATT	0.993	0.938–0.999	0.20	0.57
Medium-force TATT	0.995	0.951–0.999	0.16	0.44
High-force TATT	0.991	0.923–0.999	0.27	0.76

Abbreviations—ICC_3,1_: Intraclass correlation coefficient; 95% CI: 95% confidence level; SEM: standard error of measurement; MDC_95_: minimum detectable change at 95% confidence level; TATT: tibiotarsal axial traction technique.

**Table 2:** Subject demographic characteristics.

	Mean ± SD or n (%)
Age (year)	24.3 ± 5.6
Gender	
Men	8 (53.3%)
Women	7 (46.7%)
Lower Extremity	
Right	15 (50%)
Left	15 (50%)
Height (cm)	173.8 ± 11.0
Weight (kg)	69.0 ± 12.2
BMI (kg/m^2^)	22.7 ± 3.1

Abbreviations: SD, standard deviation; cm, centimeter; kg, kilogram; BMI, body mass index; n, number.

**Table 3:** Outcomes of axial movement of talus bone for magnitude of force applied with TATT, with and without tibial fixation.

Magnitude of TATT Force	Tibial Fixation	Non-Tibial Fixation	Mean Difference (95% CI)	ES	*p*-Value
Low-force (50.41 ± 11.8N)	1.27 ± 0.59 mm	0.90 ± 0.39 mm	0.38 mm (0.131; 0.623) *p* = 0.004	0.74	F = 102.693 *p* < 0.001
Medium-force (145.2 ± 24.8N)	2.75 ± 0.81 mm	1.33 ± 0.60 mm	1.43 mm (1.058; 1.797) *p* < 0.001	1.99	
High-force (190.1 ± 27.6N)	3.62 ± 0.81 mm	1.63 ± 0.66 mm	2.00 mm (1.615; 2.379) *p* < 0.001	2.69	

Abbreviations: TATT, tibiotarsal axial traction technique; N, Newtons; mm, millimeters; ES, effect size.

**Table 4:** The reliability of the application of the TATT at the different magnitudes of force in Newtons.

Magnitude of Force	ICC_3,1_	95% CI	SEM	MDC_95_
Low-force TATT	0.746	0.041–0.916	3.48	9.65
Medium-force TATT	0.884	0.770–0.944	4.56	12.63
High-force TATT	0.919	0.839–0.961	3.80	10.53

Abbreviations—ICC_3,1_: Intraclass correlation coefficient; 95% CI: 95% confidence level; SEM: standard error of measurement; MDC_95_: minimum detectable change at 95% confidence level; TATT: Tibiotarsal axial traction technique.

## Data Availability

The data presented in this study are available on request from the corresponding author.
